# Supplementary material for: Inhibition of MELK Protooncogene as an Innovative Treatment for Intrahepatic Cholangiocarcinoma
Source: Medicina (Kaunas). 2019 Dec 18;56(1):1. doi: 10.3390/medicina56010001 (PMC7023300; doi:10.3390/medicina56010001)
Supplement: Supplementary file 1 [file medicina-56-00001-s001.pdf]

SUPPLEMENTARY INFORMATION

The analysis included data from 52 patients with human cholangiocarcinoma (39 with survival data). The variables have been analysed using the Statistical Package for Social Science (SPSS, version 16.0, Chicago, IL, USA).

Descriptive statistics

**Sex**	**Number (%)**	**Mean survival (SD)**
Male	24 (61.5)	27.21 (16.49)
Female	15 (38.5)	26.47 (16.56)
Total	39	26.92 (16.30)

Mean survival is 26.92 months (SD 16.30)

**Sex**	**Number (%)**	**Mean MELK mRNA (SD)**	**Mean FOXM1 mRNA (SD)**	**Mean EZH2 mRNA (SD)**
Male	31 (59.6)	0.309 (0.138)	0.218 (0.097)	0.205 (0.091)
Female	21 (40.4)	0.267 (0.114)	0.200 (0.079)	0.175 (0.071)
Total	52	0.292 (0.129)	0.211 (0.090)	0.193 (0.084)

MELK mRNA in cholangiocarcinoma

Low and high *MELK mRNA* values were recoded into binary variables (0/1) using the median values (MELK mRNA = 0.271) as cut-off. The whole dataset was then divided into 23 subjects with values of MELK mRNA below the median, and 16 subjects above the median. Statistical comparison between the two groups was performed using the **log–rank test**:

**Marker**	**Number of subjects (%)**	**Mean survival in months (SD)**	**Log-rank test**
MELK mRNA < 0.271	23 (58.9)	35.70 (14.30)	–
MELK mRNA ≥ 0.271	16 (41.1)	14.31 (9.26)	<0.0001
Total	39	26.92 (16.30)	

Conclusion 1: *patients with MELK mRNA values beyond the median 0.271* ***survive on average shorter*** *than patients with MELK mRNA below 0.271.*

FOXM1 mRNA in cholangiocarcinoma

Low and high *FOXM1 mRNA* values were recoded into binary variables (0/1) using the median values (FOXM1 mRNA = 0.221) as cut-off. The whole dataset was then divided into 20 subjects with values of FOXM1 mRNA below the median, and 19 subjects above the median. Statistical comparison between the two groups was performed using the **log–rank test**:

**Marker**	**Number of subjects (%)**	**Mean survival in months (SD)**	**Log-rank test**
FOXM1 mRNA < 0.221	20 (51.3)	36.60 (15.94)	–
FOXM1 mRNA ≥ 0.221	19 (48.7)	16.74 (8.90)	<0.0001

Total	39	26.92 (16.30)	

Conclusion 2: *patients with FOXM1 mRNA values beyond the median 0.221* ***survive on average shorter*** *than patients with FOXM1 mRNA below 0.221.*

EZH2 mRNA in cholangiocarcinoma

Low and high *EZH2 mRNA* values were recoded into binary variables (0/1) using the median values (EZH2 mRNA = 0.186) as cut-off. The whole dataset was then divided into 20 subjects with values of EZH2 mRNA below the median, and 19 subjects above the median. Statistical comparison between the two groups was performed using the **log–rank test**:

**Marker**	**Number of subjects (%)**	**Mean survival in months (SD)**	**Log-rank test**
EZH2 mRNA < 0.186	20 (51.3)	38.85 (13.62)	–
EZH2 mRNA ≥ 0.186	19 (48.7)	14.37 (6.41)	<0.0001
Total	39	26.92 (16.30)	

Conclusion 3: *patients with EZH2 mRNA values beyond the median 0.186* ***survive on average shorter*** *than patients with EZH2 mRNA below 0.186.*

Multivariate Cox regression analysis

A multivariate Cox proportional hazard model was constructed with survival as the outcome variable. The three predictors, MELK, was included, and subsequently all the variables have been entered together (full model). Hazard ratios (HRs) and their 95% confidence intervals were calculated, and the Wald test was used for model testing.

**Covariates**	**Full model (HR and 95% CI)**	**Full model (HR and 95% CI)**	**Full model (HR and 95% CI)**
Age	0.998 (0.963–1.034)	0.949 (0.912–0.987)*	1.011 (0.978–1.046)
Male sex	1.121 (0.440–2.855)	3.647 (1.362–9.768)*	2.209 (0.909–5.367)
Cirrhosis (y/n)	0.958 (0.300–3.060)	0.868 (0.296–2.548)	1.653 (0.597–4.578)
Etiology			
HBV	Reference	Reference	Reference
HCV	8.415 (1.633–43.36)*	2.084 (0.495–8.766)	1.829 (0.385–8.694)
Hepatolithiasis	3.066 (0.592–15.89)	3.783 (0.832–17.19)	3.233 (0.695–15.03)
PSC	22.42 (1.115–451.1)*	64.90 (3.951–1066.1)*	45.10 (2.450–830.5)*
Diameter > 5 cm	2.525 (0.858–7.433)	1.280 (0.427–3.835)	0.901 (0.340–2.386)
Lymph node metastasis	2.377 (0.843–6.697)	3.608 (1.254–10.38)	1.196 (0.456–3.135)
Differentiation			
Well	Reference	Reference	Reference
Moderately	1.110 (0.235–5.237)	5.498 (1.135–26.62)	1.142 (0.298–4.374)
Poorly	1.435 (0.346–5.945)	4.244 (1.539–11.71)	2.142 (0.884–5.187)
Tumor number			
Single	Reference	Reference	Reference
Multiple	3.448 (0.774–15.37)	1.902 (0.449–8.054)	2.623 (0.659–10.44)
MELK mRNA ≥ 0.271 (median value)	15.251 (3.775–61.619)**	–	–

FOXM1 mRNA ≥ 0.221 (median value)	–	19.554 (5.101–74.96)**	–
EZH2 mRNA ≥ 0.186 (median value)	–	–	19.676 (5.883–65.80)**

*$p<0.05$; **$p<0.0001$

Conclusion: ***Levels of MELK mRNA, FOXM1 mRNA and EZH2 mRNA greater than the medians are significant predictors of mortality in patients with cholangiocarcinoma.***
